# The HOPE (Helping to Outline Paediatric Eating Disorders) Project: development and debut of a paediatric clinical eating disorder registry

**DOI:** 10.1186/2050-2974-1-30

**Published:** 2013-08-12

**Authors:** Hunna J Watson, Julie McCormack, Kimberley J Hoiles, David Forbes, Julie Potts

**Affiliations:** 1 Eating Disorders Program, Princess Margaret Hospital for Children, Perth, Australia; 2 Centre for Clinical Interventions, Perth, Australia; 3 School of Paediatrics and Child Health, The University of Western Australia, Perth, Australia; 4 School of Psychology and Speech Pathology, Curtin University, Perth, Australia

**Keywords:** Adolescent, Child, Cohort, Eating disorder, Paediatric, Registry

## Abstract

**Background:**

The HOPE (Helping to Outline Paediatric Eating Disorders) Project is an ongoing registry study made up of a sequential cross-sectional sample prospectively recruited over 17 years, and is designed to answer empirical questions about paediatric eating disorders. This paper introduces the HOPE Project, describes the registry sample to-date, and discusses future directions and challenges and accomplishments. The project and clinical service were established in a tertiary academic hospital in Western Australia in 1996 with a service development grant. Research processes were inbuilt into the initial protocols and data collection was maintained in the following years. Recognisable progress with the research agenda accelerated only when dedicated research resources were obtained. The registry sample consists of consecutive children and adolescents assessed at the eating disorder program from 1996 onward. Standardised multidisciplinary data collected from family intake interview, parent and child clinical interviews, medical review, parent, child and teacher psychometric assessments, and inpatient admission records populate the HOPE Project database.

**Results:**

The registry database to-date contains 941 assessments, of whom 685 met DSM-IV diagnostic criteria for an eating disorder at admission. The majority of the sample were females (91%) from metropolitan Perth (83%). The cases with eating disorders consist of eating disorders not otherwise specified (68%), anorexia nervosa (25%) and bulimia nervosa (7%). Among those with eating disorders, a history of weight loss since illness onset was almost universal (96%) with fear of weight gain (71%) common, and the median duration of illness was 8 months.

**Conclusions:**

Over the next five years and more, we expect that the HOPE Project will make a strong scientific contribution to paediatric eating disorders research and will have important real-world applications to clinical practice and policy as the research unfolds.

## Background

Eating disorders are serious mental illnesses involving extreme disturbances in eating attitudes and behaviour. The lifetime prevalence of paediatric eating disorders is 0.3% for anorexia nervosa (AN) and 0.9% for bulimia nervosa (BN) [1], though overall prevalence is higher, given >50% present with “eating disorders not otherwise specified” (EDNOS) [2]. Approximately 1.1 in 100,000 children are admitted annually to inpatient eating disorder services, where life-preserving medical intervention is the focus [3]. Among consecutively admitted paediatric eating disorder inpatients, 61% had life-threatening complications of malnutrition, 58% required nasogastric feeding to preserve life, 67% met the psychological diagnostic criteria for AN, and 34% received psychotropic medications [3]. The extreme physical and psychological toll of these illnesses is reflected in the heightened risk of mortality [4] and medical consequences such as impaired vertical growth, cardiac complications (i.e., arrhythmias), gastrointestinal problems, and poor bone health (i.e., osteoporosis) [5,6]. Socioeconomic impact is high but not widely quantified; in Australia eating disorders are estimated to account for $69 billion per year [7].

Despite the serious nature of eating disorders, they are not well understood. In the past century many myths have flourished [8,9], characterising eating disorders as; “a lifestyle choice”, a “phase”, “about vanity”, diseases of “white upperclass females”, prevalent only in Western cultural environments, not posing medical risk, not occurring in people who are normal weight or overweight, a consequence of parenting, and incurable. Scientific evidence has been instrumental in overturning myths and generating break-throughs in prevention, health care, and public health policy, yet research still lags behind patient, family, health care, and public needs [10,11].

Novel investigations using a range of research methods are required to advance knowledge and registry and cohort studies based upon clinic populations and medical records are perhaps an underutilised source of information. Grey areas, within diagnosis and assessment, risk and etiological candidates, medical issues, patient and family needs, can conceivably be brought to light through these research designs. At project inception in 1996, eating disorders in children and adolescents were barely described and no evidence-based treatments were available, so the broad aim of the “HOPE Project” was to systematically describe and understand paediatric eating disorders. Nearly 20 years later, the scientific environment has progressed, however the original aims still apply; to cultivate discovery of new knowledge about paediatric eating disorders that will be of interest to the general community, health professionals, policy-makers, and individuals affected by eating disorders.

Currently few large (case *N* > 100) paediatric eating disorder registry or cohort studies exist; a review on outcomes which contained a systematic search of studies between 1980 to 2005 with observational designs, either cohort or case series, identified only one; the International Collaborative Outcome Study of Eating Disorders in Adolescence (ICOSEDA) [12-14]. Expert sites that conduct observational studies on naturalistic clinic samples are rare. Stanford University [15-17], which has a large retrospective cohort (~1,400 patients) and a prospective cohort (~160 patients) and the University of Chicago (UoC), [2,18,19] which has a retrospective cohort (~400 patients) and a prospective cohort (~200 patients) are recent exceptions. The Children’s Hospital at Westmead and the Royal Children’s Hospital in Australia are collecting prospective data and will emerge as other nuclei. The contributions these research sites have made are varied, as reflected in the citations, to summarise would prove unwieldy. On the basis of published outputs, peers have deemed the findings significant, and the registries have been complementary in knowledge generation rather than duplicative.

In other clinical areas, registry studies have been more utilised than they have in mental health and behavioural science, cancer and heart and blood diseases are examples [20]. Challenges of registry research are that the registry population is not randomly sampled, and patients who do not present for treatment or who visit service providers not participating in the registry are missed. The time and effort required to record information on standardised research forms, and obtain and archive written informed consent, can be onerous for busy clinicians. Compellingly, the samples are typically heterogenous and recruited without strict inclusion criteria. The typically wide array of data allows for flexibility in addressing evolving empirical questions and sample size is usually large compared with stand-alone studies. Finally, the design offers flexibility in the clinical research setting, where sometimes meeting the deadlines of stand-alone studies funded by granting bodies that require unique data collections and entirely new ethics approvals is difficult. We chose a registry design because of the possibilities and flexibility - registry studies offer the opportunity to answer large numbers of questions efficiently and cost effectively and offer the best method to study the natural history of diseases, especially rare ones in real world clinical environments with heterogeneous patient groups [21]. There can be specific advantages when a priori hypotheses are difficult to define, as was the case when the HOPE Project team commenced this research.

This paper introduces the HOPE Project, a large cross-sectional registry containing data of sequential participants recruited over 17 years in a specialist paediatric tertiary eating disorder program. The HOPE Project registry is a newly available research resource for paediatric eating disorders, which aims to address evolving scientific questions. The main objective of this report is to outline the development and methodology of the HOPE Project registry, and to profile in brief the baseline psychosocial, clinical, and medical characteristics of the sample to-date. Secondary objectives are to communicate our research strategic plan and to describe the lessons learned developing this registry within a traditional clinical setting.

## Methods

### Registry sample

The registry (*N* = 941) comprises 194 children (< 13 years) and 747 adolescents (13–17 years) consecutively assessed by a specialist eating disorder program at a tertiary academic hospital from service inception (1996) to April 2013; though the registry is ongoing. We define children as < 13 years because the average age of menarche onset is 12.8 years [22]. Referrals inclusive to age 16 are currently accepted and prior to 2005 referrals to age 17 inclusive were accepted because of a public health service gap.

At the time of writing, the HOPE Project had an inclusion rate of 96.7%. All patients (and families) assessed by the Princess Margaret Hospital for Children Eating Disorders Program (PMH EDP) historically since inception to 2012 were included in this study; except for a small percentage (*n* = 27) who did not want their information used for quality and research purposes. Therefore, the inclusion rate from 1996–2012 was 97%. In 2013 a modified consent processes was introduced and 20/25 (80%) of invited patients and families participated.

Ethics approval for the current iteration of the HOPE Project was granted by PMH Human Research Ethics Committee (HREC). HREC support occurred over many years; leading toward approval for the iteration we now call the HOPE Project. Various consent procedures were used historically, due to different requirements for ethics over this time period and a registry specific waiver of new consent was granted for the recent iteration of the project, which gained approval in 2012. Currently we are trialling a new consent process with opt-in parental consent, patient assent, and patient reconsent at age 18 years, which may attenuate the inclusion rate going forward.

### Clinical setting

PMH EDP is the clinical setting and operates the only public paediatric eating disorder program in the state. Referrals are accepted from general practitioners, paediatricians, psychiatrists, psychologists, school nurses and counsellors, other professionals, and the general public. There are no definitive service exclusion criteria, although there is initial diversion towards community services if no indicators for medical admission are present, weight loss is not sustained or rapid, and the family are able to engage with community services. Patients for whom community treatments do not avert decline can be re-referred. PMH EDP is multidisciplinary with staff spanning paediatric medicine, psychiatry, clinical psychology, nursing, dietetics, occupational therapy, physiotherapy, social work, and teaching staff. Historically, PMH EDP provided a small clinical service focused on outpatient care with short medical admissions. With expanded funding which saw a ten-fold increase in clinical staffing in 2007, the service evolved to offer an integrated and more comprehensive continuum of care ranging from outpatient to day patient and inpatient, with outreach support plus a statewide training and consultation program. Research also had the opportunity to flourish as detailed next.

### Study background

PMH EDP clinical service was established initially with a service development grant, with a strong research ethos enabling data collection on all assessments from service inception. Despite minimal research resources and a commanding period of clinical expansion in the years following, a strong research culture continued; data collection remained unchanged, with the exception of adding or changing measures. The significant milestones in progressing the registry research agenda were the funding of part-time researchers through project grants in 2008- and 2010–2011 respectively, which enabled electronic input of hard copy data and introduction of a follow-up assessment clinic (2011-) in collecting 6-, 12-, and 24-month data. Currently, 65 participants (7.2%) have attended the 6 month follow-up clinic, whilst 28 participants (3.1%) have attended the 12 month follow-up clinic. The 24 month follow-up clinic will begin in 2013. During the period of funding expansion a permanent part-time research position was developed and recent grant acquisitions have secured ongoing research capacity. This has led to the completion of electronic data input, data cleaning, a research strategic plan, and capacity for research support to local universities. Historical inpatient records that date back to the beginning of the registry have also been acquired for the HOPE Project.

All data were collected prospectively on standardised research forms; thus none of the data were collected retrospectively (i.e., through chart reviews). From inception, data have been collected through two means, through standardised clinical and research instruments completed by the clinician, parents, child, and/or teacher. The second means is via research coding boxes (developed by clinical researchers) that appear on standard clinical forms completed during routine assessment. Hence, data for the cohort are prospective and standardised; the exceptions are the diagnostic, amenorrhoea, and EDE dissatisfaction variables that underwent cleaning (described later) and the body mass index (BMI) *z* score which underwent updating (described later), although based entirely on prospectively collected source data.

### Procedure

**Figure 1:**
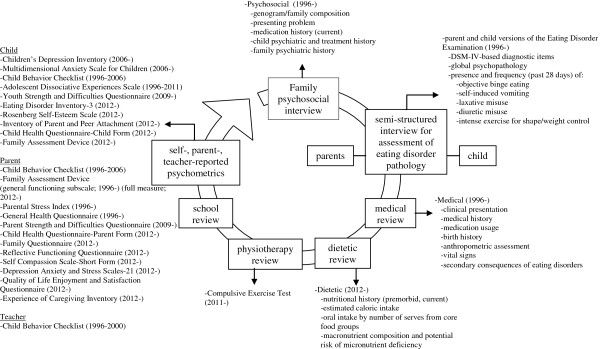
Routine intake assessment within the eating disorder service.

Data were abstracted from a clinical audit database based on the data collection process described and from research forms stored in the medical and psychological records where data were found to be missing during audit. All intake data originated from routine intake assessment (see Figure 1) spanning two consecutive half-days. Sociodemographic and psychosocial characteristics were abstracted from the psychosocial interview record form and psychometric assessments, which measure issues related to the presentation (i.e., comorbid psychopathology) plus broader areas such as parental stress and family functioning. Clinical characteristics at intake and lifetime clinical characteristics (i.e., duration of illness, previous eating disorder treatment, current suicidal ideation) were abstracted from the record forms of the psychosocial interview, medical review, and the separate child and parent versions of the clinician-administered Eating Disorder Examination interviews [23], and psychometric instruments. Medical variables were abstracted from the medical review record form. Six and 12-month follow-up data has likewise been extracted from routine follow-up assessment, with 24-month data upcoming. All variables were inputted by research staff, trained students, or research assistants.

Eating disorder diagnostic status was established with two respective nomenclatures, the fourth edition of the Diagnostic and Statistical Manual (DSM-IV) [24] and the tenth revision of the International Classification of Diseases (ICD-10) [25]. Diagnostic status with respect to an eating disorder was originally inputted into the registry database based on the DSM-IV diagnosis assigned by the clinical team at a multidisciplinary assessment meeting, which is recorded for each patient on his/her psychosocial interview form along with other summary data, clinical formulation, and treatment plan. However, during audit where error and bias were noted, diagnoses for each case were re-assigned retrospectively (discussed later).

### Quality assurance

To reduce bias in clinical diagnosis and account for changes in diagnostic criteria and systems over time, the diagnoses of all individuals in the HOPE Project registry were reviewed in 2012. To this end, the standardised medical questionnaire and child Eating Disorder Examination (EDE) [23] form, both administered at intake, were reviewed, and a research diagnosis was assigned. A hierarchical diagnostic scheme was applied whereby individuals were evaluated for AN, BN, and EDNOS/atypical eating disorders. Pertinent barriers to reliable archival diagnostic abstraction for the entire registry from clinical team assessment, and that led to the decision to re-assign intake diagnosis were: diagnostic status at intake was absent for some cases (sometimes clinicians confirmed diagnosis in the first three months as more information became available), ICD and DSM were interchangeably reported, and occasional errors in diagnostic records were apparent, for instance, a child who approximated but did not meet threshold diagnostic criteria for AN or BN may have been ascribed one of these diagnoses; while clinically some degree of flexibility is appropriate this does not satisfy the methodological rigour necessary for research.

Some systematic errors in measurement were identified and specific correction remedies followed. Amenorrhoea was extracted from the medical questionnaire but oral contraceptive (OC) use was not recorded on the medical research form. An audit of child and parent EDE records, which included an item pertaining to OC use, enabled restriction of amenorrhoea coding to the subpopulation of female patients without OC pill use. Some clinician coding errors became apparent during data entry of the EDEs, identifiable because detailed note-taking on the EDE record form by the administering clinician is positioned alongside the research coding boxes. Occasionally, administering clinicians counted intense exercise episodes/duration that was for reasons other than weight and shape control (i.e., social) within the scoring of the exercise variables. When rating dissatisfaction with shape and body weight, sometimes patients endorsed dissatisfaction in a healthy direction (i.e., dissatisfied “because I am still too bony and want to look healthier”), which instead of leading to a high score should be scored zero by the administering clinician. These issues prompted an audit of all child and parent EDEs by research assistants, and recoding of scores where necessary. We audited male records carefully during this process, checking that dissatisfaction items were scored in the appropriate direction, with discussions with a senior clinical staff member (J. M.) to establish consensus where necessary. All staff receive training on the EDE, prior to administration and we have also carried out sessions with staff on EDE scoring, and individualised staff feedback via an audit of EDE measure completion. Lastly, only one investigator (K. H.) familiar with these issues is permitted to enter EDE data.

To recover missing information, we exhaustively audited all patient files, searched all storage spaces, and searched for archived data on the hospital’s electronic records management services.

The Centers for Disease Control and Prevention revised and updated their growth charts in 2000, thus all BMI *z* scores were later audited and updated.

### Measures

The HOPE Project has involved the administration of several well-recognised psychometric instruments and other study-specific standardised research record forms over time (see Figure 1 for measures and time periods of data collection). For brevity, below we report on the psychometric instruments and measures that contributed data to the present report. Further general information about study-specific measures is deferred to Figure 1.

#### Sociodemographics

Sociodemographic data, including sex, age, rural residence, and parents at residence, were recorded by a clinician during the psychosocial intake interview with parents and child.

#### Eating disorder examination

Eating disorder symptoms were assessed via clinician-administered structured interviews; the child and parent-informant versions of the EDE [23], respectively. The EDE was originally designed for adults and is widely considered the gold standard for assessing eating disorder pathology and assisting to yield diagnosis. The EDE provides a global rating of eating disorder psychopathology and frequencies of disordered eating behaviours, including objective binge eating, self-induced vomiting, excessive exercise for shape and weight control, and laxative and diuretic misuse, over the previous 28 days. Cognitive symptoms, such as fear of weight gain, are measured by subscale items.

#### Percent expected BMI and BMI *z* score

Children were weighed wearing underwear and a hospital gown, using electronic scales accurate to 50 gm. Height was measured with the patient barefoot standing on a hard surface and using a Hardpenden stadiometer, accurate to 1 mm. Percent expected body mass index (BMI kg/m^2^) based on Centers for Disease Control 2000 growth charts demarcated children with a BMI < 85% of expected. BMI *z*-scores were calculated via Epi Info 7 [26] by entering the patient’s age, height, and weight into the program.

#### Depression, anxiety, and suicidal ideation

Depression symptoms were assessed with the Children’s Depression Inventory (CDI; clinical cut-off: *T*-score ≥ 65) [27] and anxiety symptoms with the Multidimensional Anxiety Scale for Children (MASC; clinical cut-off: *T*-score ≥ 65) [28]. Current suicidal ideation was recorded if the child endorsed at least one of the two responses on item 9 on the CDI (i.e., “I think about killing myself but I would not do it”, “I want to kill myself”).

#### Other clinical information

Family history of an eating disorder was assessed during the psychosocial interview by asking parents whether a relative has had a past or current eating disorder. The response (yes/no) and type of disorder, if affirmative, were recorded by the clinician. This method is unvalidated and has unestablished reliability and validity. Duration of illness was recorded on the medical questionnaire by the physician at medical review and was obtained through discussion with the parents and child present. The physician recorded maximum previous weight to determine history of weight loss. Amenorrhoea was defined in accordance with DSM-IV anorexia nervosa criteria and was coded based on the physician’s classification of the individual as amenorrhoeic, though later cleaned to exclude cases taking the OC pill (described earlier).

## Results and discussion

At the time of this report, the HOPE Project registry database contains 941 youth referred and assessed. Of the 941, 685 met criteria for a DSM-IV eating disorder, 168 did not, and for 88, eating disorder status was unable to be determined due to missing height/weight data (*n* = 3) or child EDE interview records (*n* = 85). Only one critical item needed to be missing to render the case unsuitable for confirmation of research diagnosis.

The profile of the cohort is shown in Table 1. Most patients were female, adolescent, and had a diagnosable eating disorder. According to medical records and clinical observation, the cases without formal eating disorder diagnosis (*n* = 168) commonly had unusual food habits, related to other psychiatric diagnoses such as obsessive-compulsive disorder, other anxiety disorders, depression, problems such as picky or selective eating, or physical problems such as chronic fatigue syndrome, irritable bowel syndrome, Crohn’s disease, celiac disease, or, in rare cases, a brain tumour. Ego-syntonicity and lack of endorsement of cognitive symptoms led to some not meeting diagnostic criteria, with ongoing engagement with PMH EDP suggesting eventual clinical diagnosis. Other assessed children were symptomatic (i.e., severe body image concerns) but missed the diagnostic thresholds; according to clinical literature [29] these children may have been prodromal, subclinical, at outer limits of normal parameters, or syndromal but casualties of the limitations of the DSM-IV taxonomic system especially as it applies to children. We plan to investigate these diagnostic issues further outside of this report.

**Table 1:** **Sociodemographic characteristics of the entire cohort (*N*** **= 941) and eating disorder status**

	***N***	**Entire cohort**
Sex (female)	941	852 (90.5%)
Age, *M* ± *SD* [range]	941	14.34 ± 2.02 [4.25,17.92]
<12 yrs		111 (11.8%)
12-14 yrs		207 (22.0%)
14- 16 yrs		455 (48.4%)
>16 yrs		168 (17.9%)
Rural residence	915	152 (16.6%)
Parents at residence	822	
Biological parents		531 (64.6%)
Mother and stepfather		149 (18.1%)
Single mother		112 (13.6%)
Other		30 (3.6%)
Eating disorder status	941	
No eating disorder		256 (27.2%)
DSM-IV eating disorder		685 (72.8%)

Estimates are *n* (%) unless otherwise indicated. *N* column is total number with valid (i.e., non-missing) data.

The profile of the subset of the cohort with eating disorders is shown in Table 2. Most patients in the HOPE Project registry met the criteria for EDNOS. On average, patients presented at approximately 14 years of age with a recent history of weight loss. Intense exercise (45%), self-induced vomiting (28%), and objective binge eating (19%) were commonly reported disordered eating behaviours. Similar to observations in other paediatric studies [16], laxative and diuretic misuse among children and younger adolescents is rare, perhaps because these are harder to access for this age group and require financial resources. Also prevalence may be higher than self-report, with one previous study among adolescents with AN at our service finding that prevalence of laxative use increased almost two-fold relative to self-report when positive screens from biochemical tests were counted [30].

**Table 2:** **Sociodemographic, clinical, and medical characteristics of the eating disorder subset of the cohort (*n*** **= 685)**

	***N***	**Eating disorder clinic cohort (*N*** **= 685)**
**Sociodemographic characteristics, total sample**		
Sex (female), *n* (%)	685	641 (93.6%)
Age, *M* ± *SD* [range]	685	14.7 ± 1.6 [8.7,17.9]
<12 yrs		45 (6.6%)
12-14 yrs		146 (21.3%)
14-16 yrs		374 (54.6%)
>16 yrs		120 (17.5%)
Rural residence, *n* (%)	663	115 (17.3%)
**Family history, total sample**		
Eating disorder history, *n* (%)	572	151 (26.4%)
**Physical features, total sample**		
BMI *z* score, *M* ± *SD* [range]	636	−1.41 ± 1.48 [−8.17,2.13]
Weight loss^1^, *n* (%)	538	514 (95.5%)
< 85% of expected height/weight, *n* (%)	577	355 (61.5%)
Amenorrhoea, *n* (%)	659	280 (42.5%)
Primary		30 (4.6%)
Secondary		250 (37.9%)
**Comorbid psychopathology, total sample**		
CDI *T*-score ≥ 65, *n* (%)	262	127 (48.5%)
MASC *T*-score ≥ 65, *n* (%)	264	89 (33.7%)
Current suicidal ideation, *n* (%)	271	114 (42.1%)
Current self-harm, *n* (%)	493	134 (27.2%)
**Eating disorder features, total sample**		
Duration of illness (months), median ± IQR [range]	635	8 ± 5–12 [1,120]
Diagnosis, *n* (%)	685	
Anorexia nervosa		172 (25.2%)
Restricting		141 (20.6%)
Binge/purge		31 (4.5%)
Bulimia nervosa		48 (7.0%)
Purging		45 (6.6%)
Non-purging		3 (0.4%)
Eating disorders not otherwise specified		465 (67.9%)
Child version: EDE global, *M* ± *SD* [range]	675	3.07 ± 1.57 [0.0,5.95]
Parent version: EDE global, *M* ± *SD* [range]	598	2.94 ± 1.29 [0.0,6.93]
Objective binge eating, *n* (%)	685	129 (18.8%)
Purging, *n* (%)	685	237 (34.6%)
Self-induced vomiting, *n* (%)	685	195 (28.5%)
Laxative misuse, *n* (%)	685	49 (7.2%)
Diuretic misuse, *n* (%)	685	5 (0.7%)
Intense exercise to control shape/weight, *n* (%)	685	307 (44.8%)
Fear of weight gain (≥ 4 on EDE item), *n* (%)	673	479 (71.2%)
**Eating disorder features, those with behaviour present**		
Objective binge eating, median ± IQR [range]	129	10 ± 3–19 [1,168]
Purging	237	20.00 ± 5–33.5 [1,308]
Self-induced vomiting, *M* ± *SD* [range]	195	27.17 ± 32.07 [1,224]
Laxative misuse, *M* ± *SD* [range]	49	22.29 ± 47.13 [1,280]
Diuretic misuse, *M* ± *SD* [range]	5	50.40 ± 23.43 [28,84]

Discrepancies in available *N* on the CDI and MASC reflect the later introduction of these measures. Values are *n* (%) unless otherwise indicated. Median and IQR are presented for skewed variables. *BMI* body mass index, *CDI* Children’s Depression Inventory, *EDE* Eating Disorder Examination, *MASC* Multidimensional Anxiety Scale for Children. ^1^Lower weight at intake than parent- or self-reported maximum ever weight.

Although psychiatric comorbidities are not formally diagnosed in the HOPE Project sample, symptoms of depression and anxiety were judged on dimensional rating scales and found to be common, particularly depressive symptoms and current suicidal ideation. Comorbidity of mood and anxiety symptoms is well-established in adults with eating disorders [31] and there is recognition of these relationships among young people too, particularly depression [16,32].

The geographic spread of referrals was evident, with 17% (1 in 6) referred from rural Western Australia (up to four days drive away from PMH EDP). Rural-based referrals may be underrepresented at admission; compared to Western Australia population estimates, 27% of persons aged 5–19 years reside in rural Western Australia [33]. Alternatively in the absence of national or state epidemiological data, there may be an unknown difference in prevalence rates in rural and metropolitan Australia.

General comparability of the HOPE Project sample is evident with the UoC (*N* = 401) [18] and Stanford (*N* = 1432) [15] observational study samples. All contain children and adolescents consecutively referred to a specialist eating disorder program, Stanford and HOPE Project participants were drawn from outpatient to inpatient care, and UoC from outpatient. Like the other samples, ours contains mainly females (HOPE 94%; Stanford 91%; UoC 90%) in mid-adolescence (HOPE *M* = 14 years, UoC *M* = 15 years, Stanford *M* = 15 years), with predominantly EDNOS (HOPE 68%; UoC 60%; Stanford 63%) and an approximate one year duration of illness (HOPE median = 8 months; Stanford *M* = 15 months; UoC not reported). Difficulty lies in comparing the HOPE profile to other paediatric eating disorder samples in the scientific literature, for many well-characterised groups have been randomised controlled trial (RCT) samples or based on small retrospective studies that restrict diagnosis to certain eating disorders (i.e., [34]). There may be some systematic differences between RCT and clinic treatment seeking-youth samples [35]. A strength of the HOPE Project is that sampling is naturalistic and ecologically representative of a tertiary setting. Selection bias is likely low, given the sequential recruitment method, lack of exclusion criteria, variety of service referral methods, and large catchment area. The service is the only specialist public (free-of-charge) paediatric eating disorder service in Western Australia (which has a total population of 2.45 million) [36] and the primary port of tertiary referral for health professionals and other members of the community. The very high inclusion rate negates selection bias, though biases may become apparent in future studies when missing data patterns are evaluated.

The cohort would be expected to be dynamic over time, reflecting changing trends in epidemiology and state health system capacity, which will be explored in subsequent HOPE studies. During the lifespan of the HOPE Project, there has been some evidence that the prevalence of eating disorders has continued to increase [37] while the age of onset of eating disorders has decreased [1], and greater recognition of eating disorders and burden and severity [8]. Whether these trends hold true is impossible to know, accurate descriptions of epidemiology across nations and including EDNOS has only just recently been described and replication is necessary. The authors have observed that the phenomenology of eating disorders has followed the trends of eating and body concerns in society in general, for example with increasing emphasis on exercise and health, and changes in cognitive content from sugar, towards fat and more recently carbohydrates. Over the last two decades, anecdotally referrals to PMH EDP have increased with no equivalent growth in assessment numbers and greater diversion of cases to community settings, which may mean cohort patients in the latter years are a more physically or psychiatrically unwell group than for the early days of the program.

Looking forward, it is envisioned that the HOPE Project registry will produce valuable knowledge in specific areas of paediatric eating disorders, such as diagnosis and assessment, epidemiology, risk and etiological candidates, medical issues, and patient and family needs, and generate implications for policy, practice, and research. As well as enabling comparisons with findings from other cohorts, we anticipate that the large number of variables contained in the HOPE Project will significantly enable expansion of knowledge into new territories. A scoping project undertaken under the oversight of the HOPE Project steering committee has identified research priorities and possibilities for the next five years. Selection of projects follows a governance strategy that places precedence on clinically- and/or policy-relevant projects.

Three HOPE studies are underway. The first, examines parent–child concordance on the EDE, to highlight gaps and issues in parent and child reporting, which we expect to assist assessment procedures. The second, compares children and adolescents with eating disorders on cognitive, behavioural, diagnostic, and medical presentation and aims to inform health care professionals about developmental issues. The third study investigates rural health inequalities, and whether rurality translates to a more compromised clinical and medical status at presentation, and greater inpatient service use.

Several projects are in the planning phase. In the eating disorders field, there has been debate over the applicability of diagnostic systems to younger populations [38]. We plan to compare DSM-IV, proposed DSM-5 (which has also been coded in our registry), and ICD-10 diagnostic systems, to examine concordance and whether the preponderance of EDNOS diagnoses is reduced with the DSM revisions. We will also examine those who do not meet formal diagnostic criteria for an eating disorder at admission but go on to have subsequent treatment. Gender differences will be explored taking into account physical, psychological and diagnostic factors, with potentially one of the larger samples of young clinical males available, to investigate similarities and differences from females. Finally, efforts to describe and characterise patients on clinical features, such as severity dimensions or symptom clusters would assist the field to characterise the heterogeneity within eating disorder diagnoses. The cohort database may be utilised to explore, test or replicate taxonomic models of eating disorders such as the functional staging model of anorexia nervosa [39] due to its broad array of medical, behavioural, and psychological variables.

The HOPE Project is embedded within a five-year research strategic plan for the PMH EDP, which goes wider than traditional research outputs and focuses on building a dynamic, collaborative, and sustainable research team culture. Areas of emphasis include: the development of research capacity, data management infrastructure and processes, scholarly output, and partnerships, collaboration and leadership. Promoting knowledge-practice transfer both within and beyond the PMH EDP, including with consumer and carer groups, is a key goal. Other important outcomes include developing researchers and clinicians with dual expertise and commitment, increasing levels of staff participation, confidence and capacity, and developing internal mechanisms and structures for up-to-date dissemination and integration of scientific knowledge. Challenges to collaboration between clinical and research agendas include clinical pressures and a lack of confidence by clinicians in their research capabilities. Current strategies include whole-of-team research meetings that give visibility to research issues and enable sharing of knowledge and skills, joint project teams that partner researchers and clinicians, and the allocation of research time for clinicians. We have found that an agenda that balances clinical and research issues is helpful as well as prioritisation of research projects with high clinical utility and ensuring research protocols have obvious clinical relevance. We believe research questions and interpretation of findings becomes richer and more relevant when embedded in a clinical context, and whilst researchers and clinicians contribute differently to the research process, ultimately with time and mutual exchange of knowledge and skills, we anticipate the products of this collaboration will be synergistic. In the future, we plan to develop mechanisms for consumer participation in the HOPE Project that go beyond contributing personal data, and include consumers and carers in research processes such as determining study prioritisation, design, and discussion of implications.

Already we have learnt many lessons in undertaking registry research in a clinical setting, and some constant challenges remain including sourcing financial support for the ongoing registry when funders typically prefer time-limited and specific projects, and securing dedicated research roles and resources. Obtaining adequate financial support for the HOPE Project and/or follow-up protocols has so far remained elusive; however attaining distinct project funding has proved more achievable. Embedding larger data collection goals relevant to the HOPE Project within discrete projects has enabled the progression of the project, alongside the creation of dedicated research positions, creative use of student volunteers, and an arrangement with the local universities that students contribute time to data entry and cleaning in exchange for use of registry data.

Managing the potential for measurement bias (i.e., inaccuracies in data recording, missing data) is an ongoing challenge, attributed to staff flux (changeover, practicum students) and varying levels of awareness of the importance and uses of the data, and inadequate research resourcing to maintain data collation and entry activity. Using cohort data is essential in promoting the value of the database [40], and this alongside routine focus on data integrity will ensure longevity of the registry. Preventative action we have found helpful includes dedicated research roles, regular training and supervision, auditing of records and measures with confidential individualised feedback to clinicians, integration of quality and research goals, less rotation of assessment staff, and encouragement of good record-keeping skills. Quality assurance is an ongoing issue managed as part of the HOPE Project and we have found that prevention (and early intervention) is the best cure.

Resources exist for assisting eating disorder researchers to understand and avoid the pitfalls associated with controlled trials [41], yet fewer resources were identified to support ongoing observational research. HREC grant processes may need revision to ensure registry studies are not evaluated through the same lens as controlled treatment trials. There is a growing movement within epidemiology to educate HRECs about issues specific to observational studies (i.e., problem of selection bias), so that standards historically designed and applied for controlled trials (i.e., opt-in consent rather than opt-out consent) can be reconsidered in observational designs to achieve balance on research for the good of the public and the individual’s right to privacy [42,43].

## Conclusions

This paper has described and debuted the HOPE Project, an ongoing clinical registry of children and adolescents referred for eating disorder assessment at a specialist hospital-based program over a 17-year period. We believe the HOPE Project will offer a unique and ongoing opportunity to cultivate scientific knowledge about paediatric eating disorders. The trials and tribulations of establishing a research registry within an evolving clinical service have been described, and we have outlined that although challenging, research of this nature, in situ in a clinical setting, is both possible and extremely valuable. The potential for illuminating the nature of paediatric eating disorders, as they appear and evolve in an archetypal public health service, is immense. It is our aspiration that ultimately the knowledge generated from the HOPE Project will inform prevention, early intervention, assessment and treatment practices in real world clinical and policy environments.

## Abbreviations

AN: Anorexia nervosa; BMI: Body mass index; BN: Bulimia nervosa; DSM: Diagnostic and Statistical Manual; EDE: Eating Disorder Examination; EDNOS: Eating disorders not otherwise specified; HOPE: Helping to Outline Paediatric Eating Disorders; ICD: International Classification of Diseases; PMH EDP: Princess Margaret Hospital for Children Eating Disorders Program.

## Competing interests

The authors declare that they have no competing interests.

## Authors’ contributions

JM, JP, and DF enabled standardised data collection from the outset of PMH EDP inception. JM, JP, DF and HW conceived the cohort study. HW acquired ethical approval, established the cohort registry database, prepared the manuscript, and conducted data analysis. KH assists to manage the registry database and project and conducted data analysis. JM has managed the project since it was conceived, acquired research resourcing, conducted regular assessment training with staff, and prepared the manuscript. All authors reviewed and approved the final version of the manuscript.

## Authors’ information

HJW, PhD, MPsych(Clin). Senior Research Psychologist at PMH EDP, Senior Research Scientist at Centre for Clinical Interventions, Adjunct Research Fellow at the School of Paediatrics and Child Health, The University of Western Australia, and Adjunct Lecturer at the School of Psychology and Speech Therapy, Curtin University, Perth, Australia. JM, MPsych(Clin). Specialist Clinical Psychologist at PMH EDP, Perth, Australia. KH, BSc(Hons)Psych. Research assistant at PMH EDP and PhD Candidate at the School of Psychology and Speech Pathology, Curtin University, Perth, Australia. DF, MB BS. FRACP. Paediatrician at PMH EDP and Associate Professor at the School of Paediatrics and Child Health, The University of Western Australia, Perth, Australia. JP, BSc(Nurs). Manager at PMH EDP, Perth, Australia.
